# Decline of phosphatidylethanol (B‐PEth) during abstinence in patients with alcohol use disorder undergoing withdrawal treatment, and the correlation of B‐PEth with self‐reported alcohol intake

**DOI:** 10.1111/add.70359

**Published:** 2026-02-19

**Authors:** Andrea de Bejczy, Lisa Walther, Cecilia Nilsson‐Wallmark, Barbro Askerup, Anders Isaksson

**Affiliations:** ^1^ Addiction Biology Unit, Psychiatry and Neurochemistry Section, Institute of Neuroscience and Physiology, Sahlgrenska Academy University of Gothenburg Sweden; ^2^ Department of Addictions and Dependency Sahlgrenska University Hospital Sweden; ^3^ Division of Clinical Chemistry and Pharmacology, Department of Laboratory Medicine Lund University Lund Sweden; ^4^ Department of Clinical Chemistry and Pharmacology Skåne University Hospital Lund Sweden

**Keywords:** abstinence, alcohol, alcohol biomarker, alcohol use disorder (AUD), alcohol withdrawal, decline, elimination, half‐life, phosphatidylethanol (B‐PEth)

## Abstract

**Background and aim:**

Accurately estimating alcohol consumption is crucial for clinical decision‐making and monitoring treatment outcomes. Phosphatidylethanol in blood (B‐PEth), a direct alcohol biomarker, is currently the most reliable indicator of alcohol intake, with a detection window of several weeks; however, the factors influencing the decline of B‐PEth levels remain largely unknown. This study aimed to investigate the decline of B‐PEth levels during abstinence in patients with alcohol use disorder (AUD) undergoing withdrawal treatment.

**Method:**

A total of 100 patients were recruited in withdrawal treatment and followed during three to four weeks of abstinence. Blood samples were collected at baseline and weekly during abstinence to measure B‐PEth levels of two homologues (16:0/18:1 and 16:0/18:2). Self‐reported alcohol consumption was documented using the Timeline Followback (TLFB) method for 30 days before abstinence and throughout the study period.

**Results:**

B‐PEth elimination followed first‐order kinetics. The mean half‐life was 7.24 days [95% confidence interval (CI) = 6.98–7.53] for 16:0/18:1 and 4.55 days (95% CI = 4.44–4.67) for 16:0/18:2. The rate of decline varied by week, with a longer half‐life observed in week three compared with week one. No statistically significant sex differences were detected. The strongest correlation between B‐PEth levels and self‐reported alcohol consumption was found for data from two weeks prior to abstinence.

**Conclusion:**

Elimination of phosphatidylethanol in blood (B‐Peth) follows first‐order kinetics, with homologue 16:0/18:1 exhibiting a longer half‐life than 16:0/18:2. The rate of decline is influenced by the week of alcohol abstinence and B‐PEth levels are detectable even in the fourth week of abstinence. The strongest correlation between B‐PEth and self‐reported alcohol consumption is at two weeks prior to abstinence.

## INTRODUCTION

Accurately assessing alcohol consumption is essential in various medical and legal contexts, including alcohol screening, alcohol use disorder (AUD) treatment, pre‐surgical evaluations and forensic investigations.

Blood phosphatidylethanol (B‐PEth) level is increasingly recognised as the most reliable alcohol biomarker [1, 2]. It is a direct alcohol marker formed in erythrocyte membranes exclusively in the presence of ethanol, making it highly specific and sensitive [3]. B‐PEth levels reflect alcohol consumption over several weeks, with higher intake leading to higher concentrations [4, 5, 6]. This makes B‐PEth a valuable complement to markers for acute or recent intake, like breath alcohol tests, which serve as proxies for blood alcohol concentration (BAC), and ethyl glucuronide (EtG), which has a detection window of 3–5 days [7, 8]. Additionally, B‐PEth is stable in human blood and remains unaffected by factors such as sex, liver disease or hypertension [2, 9, 10, 11, 12].

Among the 48 identified B‐PEth homologues, 16:0/18:1 and 16:0/18:2 are the most prevalent in humans. The 16:0/18:1 homologue is considered a strong proxy for total B‐PEth levels [2, 3, 6, 13]. B‐PEth formation begins immediately after alcohol consumption and continues as long as ethanol is present in the bloodstream.

Studies on B‐PEth elimination in AUD patients have reported mean half‐lives of 4.0–6.1 days for total PEth [4] [14] [15]. PEth 16:0/18:1 and 16:0/18:2 have shown mean half‐lives of 6.3 and 4.6 days, respectively [15].

Studies on volunteers have found mean half‐lives of 3.0–4.6 days, with a wider range of values recorded [16] [17].

The duration of abstinence may also influence B‐PEth elimination. In a study of 11 healthy volunteers, the half‐life ranged from 4.5 to 10.0 days during the first week and from 5 to 12 days during the second week [18].

Despite its growing use, research on B‐PEth decline in AUD patients undergoing withdrawal remains limited. The decline of B‐PEth over several weeks from high B‐PEth levels in patients with AUD at alcohol cessation are still not fully studied. Factors affecting B‐PEth half‐life, including sex differences, require further investigation to improve the interpretation of test results. In the clinical situation of follow‐up treatment after withdrawal treatment, B‐PEth is a valuable tool for monitoring sobriety and adherence to treatment. However, many clinicians find it difficult to interpret B‐PEth levels regarding relevant decline. Understanding the relationships among B‐PEth elimination, alcohol consumption time frame and individual variability can enhance clinical decision‐making and assessment accuracy.

Comparing objective markers like B‐PEth with self‐reported alcohol consumption has produced mixed results [1, 19, 20, 21]. Despite these inconsistencies and the bias in subjective data, self‐reported data remain widely used in both clinical and research settings, warranting further examination of the correlation with B‐PEth levels to understand their relationship.

This study aims to improve our understanding of the factors influencing B‐PEth half‐life in patients with AUD in the weeks of sobriety after withdrawal treatment as well as the optimal B‐PEth homologue for analysis. Participants were monitored during 3–4 weeks of abstinence or until relapse. Additionally, the study investigates the optimal time frame for correlating B‐PEth levels with self‐reported alcohol consumption.

## METHODS

### Study design

This study is a descriptive, non‐interventional, clinical investigation examining the decline of B‐PEth levels in patients with AUD during and after withdrawal treatment. The study was designed to address the half‐life of B‐PEth during 3 weeks of alcohol abstinence by measuring two homologues, possible sex and individual differences, and the correlation of self‐reported alcohol intake with B‐PEth levels.

### Participants

A total of 100 patients (80 males and 20 females) aged 18–75 years were included in the study (Table 1). Inclusion criteria required participants to be diagnosed with alcohol dependence, be admitted for alcohol detoxification, have a working phone number, and be able to speak and write in Swedish. Exclusion criteria included blood infections, serious somatic diseases that could affect blood analyses or pose risks during blood sampling, and substance use disorders other than alcohol and/or nicotine. Participants were recruited from the alcohol withdrawal care unit at the Department of Addictions and Dependency, Sahlgrenska University Hospital, Gothenburg, Sweden, during 2014–2016. Each participant provided written informed consent and was enrolled within 24–72 hours of admission. Ethical approval was obtained from the Swedish Ethical Review Authority (EPM; D.nr. 179–12).

**TABLE 1:** Number of patients included in analyses from total number of patients included in the study (*n* = 100).

	Subjects at scheduled study visit	Subjects excluded owing to violation of abstinence	Samples analysed according to actual week of abstinence
Week 1 of abstinence (days 1–7)	99	–	145
Week 2 of abstinence (days 8–14)	56	5	45
Week 3 of abstinence (days 15–21)	58	14	39 (36 for 16:0/18:2)
Week 4 of abstinence (days 22–28)	31^a^	5	19 (17 for 16:0/18:2)

^a^ The small number of subjects in the analysis of the fourth week of abstinence is not associated with dropout but with a shift of subjects from the intended time frame of the third week of abstinence.

### Procedure

The study included a total of four visits over 3 weeks: visit 1 for screening and inclusion was conducted on days 2–3 of abstinence during in‐patient withdrawal treatment; visit 2 was conducted on days 6–7 (the last day of in‐patient treatment; week 1 of abstinence); visit 3 was conducted on day 14 at the clinical study site (1 week after discharge; week 2 of abstinence); and visit 4 was conducted on day 21 at the clinical study site (2 weeks after discharge; week 3 of abstinence). However, as a deviation of ±3 days per visit was allowed, some subjects carried out visit 4 in the fourth week of abstinence (after 21 days of abstinence). These subjects/samples are therefore analysed in the fourth week of abstinence. The small number of subjects in the analysis for the fourth week of abstinence did not result from dropout but from a shift of subjects from the intended time frame in the third week of abstinence. The statistical analysis is based on the actual day and not the visit number, and the data are subsequently divided into weeks of abstinence. Hence, the number of subjects at each visit will not always correlate with the number of samples analysed in each week of abstinence.

At the first visit, data on family history of alcohol problems were collected. Self‐reported alcohol consumption for the previous 30 days was assessed using the timeline followback (TLFB) method [22], along with the Alcohol Use Disorder Identification Test (AUDIT) and *Diagnostic and Statistical Manual of Mental Disorders*, Fourth Edition, Text Revision (DSM‐IV‐TR) criteria for alcohol dependence.

At every visit, the following assessments were performed: Clinical Institute Withdrawal Assessment for Alcohol (CIWA), visual analogue scale (VAS) for alcohol cravings, breath alcohol concentration (BrAC) measurement, TLFB assessment since the last visit, blood samples for B‐PEth analysis, urine samples for ethyl glucuronide (U‐EtG) analysis and drug screening. Safety analyses, including haemoglobin levels and vital signs, such as blood pressure and heart rate, were documented at all visits. The blood and urine samples were sent to a certified laboratory and stored according to standard operational procedures before being analysed consecutively within the time frame allowed for preserved quality.

After discharge, the subjects followed routine clinical out‐patient treatment at the alcohol ward with regular follow‐ups, blood sampling and pharmacological and/or psychosocial treatments, in addition to the study protocol. However, data on the subject’s individual clinical follow‐up were not included in the study protocol and are hence not available for description.

### Statistical analysis

Based on consultation with statisticians, a sample size of 100 participants was determined to be sufficient to achieve the study objectives. Data analysis was conducted by independent statisticians.

The primary outcome variables included B‐PEth levels (homologues 16:0/18:1 and 16:0/18:2) measured in blood and self‐reported alcohol consumption, recorded in grams via TLFB.

The analysis population consisted of participants who met the criteria for abstinence. Exclusion criteria for analysis included: self‐reported alcohol consumption (TLFB), objective markers of alcohol consumption, including positive U‐EtG or BrAC tests, or a rise in B‐PEth between samplings.

Statistical analysis included all available measurements from participants on abstinence days. B‐PEth kinetics were analysed based on weeks of abstinence, week 1 (days 1–7), week 2 (days 8–14), week 3 (days 15–21) and week 4 (days 22–28). Day 1 was defined as the first day of withdrawal treatment. Discrepancies in the analyses for the two homologues (Tables 2 and 3) arose from a technicality in the method of analysis, where a few samples were analysed as <0.03 μmol/l, these samples are coded as missing in the analyses (and hence are not used in the statistical analysis), owing to uncertainty of the actual B‐PEth levels (as they could be any value between 0.03 and zero).

**TABLE 2:** Descriptive characteristics and safety parameters at visit 1, days 2–3 of abstinence.

*n* (%)	Men	Women
Sex	80 (80)	20 (20)
Daily nicotine	62 (78)	13 (65)
Positive urine toxicology for drugs	8 (10)	3 (15)
Heredity for alcohol problems	61 (76)	17 (85)
**Mean (SEM); min.–max.**		
Age, years	53.7 (1.04); 27–75	49.7 (2.26); 22–64
Debut, years	15.6 (0.38); 9–29	16.2 (0.83); 11–25
Duration of alcohol abuse, years	21.3 (1.43); 0–50	16.1 (2.57); 2–40
AUDIT total score	34.5 (0.65); 7–40	35.3 (1.14); 26–40
CIWA total score	4.51 (0.30) 0–12	6.10 (0.80);0–14
Craving	71.25 (4.08); 0–100	61,9 (7.77); 0–100
Units of alcohol/day (TLFB 30 days)	14.7 (1.24); 0–60	16.3 (1.82); 2–25
Baseline B‐PEth,	2.57 (0.17); 0.45–7.45	2.25 (0.26); 0.45–4.89
Blood pressure systolic	140 (1.89); 110–180	131(3.76); 110–180
Blood pressure diastolic	91.5 (1.32); 60–120	86.5 (2.9); 65–120
Hb	148 (1.54); 101–185	135 (3.1);108–157
AST	1.32 (0.14); 0.33–7.90	1.08 (0.23);0.27–4.70
ALT	1.14 (0.94);0.24–4.10	0.83 (0.14); 0.26–2.40

Abbreviations: ALT = alanine transaminase; AST = aspartate transaminase; AUDIT = alcohol use identification test; B‐PEth = phosphatidylethanol in blood; CIWA = Clinical Institute Withdrawal Assessment of Alcohol scale; Hb = haemoglobin; *n* = number of subjects; SEM = standard error of mean; TLFB = timeline followback.

**TABLE 3:** First‐order kinetics of B‐PEth 16:0/18:1 and B‐PEth 16:0/18:2 half‐lives (*t*_1/2_, days), by sex^a^.

Variable		*n*	*t*_1/2_ (days) (95% CI)	*P*
ln(B‐PEth) 16:0/18:1	Total	100	7.24 (6.98–7.53)	N/A
ln(B‐PEth) 16:0/18:2	Total	100	4.55 (4.44–4.67)	N/A
ln(B‐PEth) 16:0/18:1	Men	80	7.17 (6.90–7.52)	0.41
	Women	20	7.45 (6.95–8.08)	
ln(B‐PEth) 16:0/18:2	Men	80	4.54 (4.41–4.67)	0.13
	Women	20	4.64 (4.39–4.91)	

Abbreviations: B‐PEth = phosphatidylethanol in blood; ln(B‐PEth) = logarithm of B‐PEth; *n* = number of subjects.

^a^ All available measurements. At abstinence week 4, interpreting half‐life at the subgroup level was not possible.

A univariate regression analysis was first conducted, with B‐PEth as the dependent variable and abstinence day as the independent variable. A mixed‐model regression analysis was then performed, using both variables as fixed effects. The decline rate of B‐PEth was estimated by fitting a regression line to individual measurements, from which individual half‐lives were calculated. As individual half‐lives were derived through linear regression, they were not identical to the model used for overall half‐life estimation. The coefficient of variation (CV) was used to measure data dispersion. The linear regression analysis is performed on a logarithm of measured B‐PEth, where actual time is used as an independent value. The regression model parameters are re‐calculated as half‐time and presented and compared. This introduced great variation and randomness in the individual values for the analysis of week 4, which renders the analysis of half‐time not feasible.

We have not found any support for the hypothesis that the dropout of patients over time is dependent on outcomes or side effects, and therefore we use the mean value over the observed follow‐up data in the analyses.

Correlation analyses were conducted using Spearman’s rank correlation test. TLFB data were categorised into four weeks prior to abstinence: week 1 (days 1–7) before abstinence; week 2 (days 8–14) before abstinence; week 3 (days 15–21) before abstinence; and week 4 (days 22–28) before abstinence.

### Analytical methods

The B‐PEth homologues 16:0/18:1 and 16:0/18:2 were determined using chromatographic methods, as described by Stenton *et al*. [23]. U‐EtG was determined via immunoassay using reagents from Ark Diagnostics, Inc. (Fremont, CA, USA), with a detection cut‐off of 0.5 mg/l.

## RESULTS

A total of 100 subjects were included in the study. The numbers of subjects/samples analysed are provided in the respective tables. The number of participants available during the 3–4 weeks of abstinence was as follows: baseline, 100; visit after first week of abstinence, 99; visit after second week of abstinence, 56; visit after third week of abstinence, 58; visit after fourth week of abstinence, 31 (Table 1). Twenty‐four subjects were excluded from the analysis owing to objective or subjective evidence of alcohol intake, as measured by U‐EtG, positive BrAC, rising B‐PEth or subjective reporting (Tables 1 and S1). The remaining dropouts arose from loss to follow‐up. The smaller number of subjects in the analysis of the fourth week of abstinence is not associated with dropout but with a shift of subjects from the intended time frame of 3 weeks of abstinence. The mean age of the included subjects was 53 years, with a male‐to‐female ratio of 80 : 20. At baseline, 75% reported daily nicotine use and 78% had a family history of alcohol problems. The average age of alcohol initiation was 15.7 years, while the mean duration of alcohol‐related issues was 20 years. The mean total AUDIT score was 35, and the mean total CIWA score was 4.83 on days 2–3 of abstinence (at inclusion). Additionally, 11% of subjects tested positive for drugs in urine screening (Table 2).

Subjects self‐reported a mean alcohol intake of 211 g (30.4–808.0 g) in the first week prior to abstinence, 192 g (0–517 g) in the second week prior to abstinence, 164 g (0–493 g) in the third week prior to abstinence and 152 g (0–493 g) in the fourth week prior to abstinence (Table S2).

The mean PEth 16:0/18:1 level was 2.17 μmol/l (2.22 μmol/l in men, 1.98 μmol/l in women) in the first week, 1.21 μmol/l (1.22 μmol/l in men, 1.09 μmol/l in women) in the second week, 0.66 μmol/l (0.64 μmol/l in men, 0.74 μmol/l in women) in the third week and 0.41 μmol/l (0.38 μmol/l in men, 0.58 μmol/l in women) in the fourth week of abstinence. Thus, the PEth levels remained detectable even after 4 weeks of abstinence.

**FIGURE 1:**
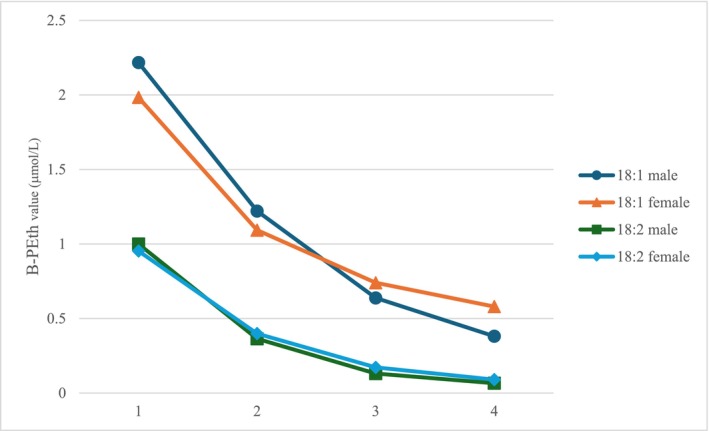
B‐PEth 16:0/18:1 and B‐PEth 16:0/18:2 mean values (μmol/L) by weeks 1–4 of abstinence and sex. B‐PEth = Phosphatidylethanol in blood.

Similarly, the mean PEth 16:0/18:2 level was 0.99 μmol/l (1.00 μmol/l in men, 0.95 μmol/l in women) in the first week, 0.37 μmol/l (0.36 μmol/l in men, 0.40 μmol/l in women) in the second week, 0.14 μmol/l (0.13 μmol/l in men, 0.17 μmol/l in women) in the third week and 0.07 μmol/l (0.07 μmol/l in men, 0.09 μmol/l in women) in the fourth week of abstinence (Figure 1; Table 4).

**TABLE 4:** B‐PEth levels (μmol/L), by week of abstinence.

	*n* ^a^	Mean (SD); min.–max.	Median
**B‐PEth 16:0/18:1**			
Week 1 of abstinence (days 1–7)	145	2.17 (1.35); 0.31–7.45	1.93
Week 2 of abstinence (days 8–14)	45	1.21 (0.68); 0.36–3.26	0.96
Week 3 of abstinence (days 15–21)	39	0.66 (0.33); 0.12–1.23	0.62
Week 4 of abstinence (days 22–28)^b^	19	0.41 (0.20); 0.05–0.75	0.41
**B‐PEth 16:0/18:2**			
Week 1 of abstinence (days 1–7)	145	0.99 (0.63); 0.15–3.56	0.87
Week 2 of abstinence (days 8–14)	45	0.37 (0.22); 0.09–0.94	0.29
Week 3 of abstinence (days 15–21)	36	0.14 (0.07); 0.03–0.31	0.14
Week 4 of abstinence (days 22–28)^b^	17	0.07 (0.03); 0.03–0.13	0.07

Abbreviations: B‐PEth = phosphatidylethanol in blood; *n* = number of measurements.

^a^ More than one measurement can be available for each subject, owing to the timing of visits.

^b^ In abstinence week 4, interpreting half‐life at the subgroup level was not possible.

The kinetics of B‐PEth followed a first‐order model, which provided the best fit with an explanatory power of 52%. In this model the half‐life of B‐PEth 16:0/18:1 during the 3‐week study period was 7.24 days (95% CI = 7.0–7.5 days) and the half‐life of B‐PEth 16:0/18:2 during this study period was shorter, at 4.55 days (95% CI = 4.44–4.67 days).

There were no significant sex differences for half‐life (*t*_₁/₂_) in B‐PEth 16:0/18:1: male *t*_₁/₂_ = 7.17 days (95% CI = 6.90–7.52 days) and female *t*_₁/₂_ = 7.45 days (95% CI = 6.95–8.08 days) (*P* = 0.41). Nor were there any significant sex differences for half‐life in B‐PEth 16:0/18:2: male *t*_₁/₂_ = 4.54 days (95% CI = 4.41–4.67 days) and female *t*_₁/₂_ = 4.64 days (95% CI = 4.39–4.91 days) (*P* = 0.13) (Table 3).

For B‐PEth 16:0/18:1, there was a significant difference in the decline across weeks: week 1 (days 1–7), *t*_₁/₂_ = 5.72 days (95% CI = 5.30–6.22 days); week 2 (days 8–14), *t*_₁/₂_ = 7.67 days (95% CI = 6.08–10.38 days) (*P* = 0.002); and week 3 (days 15–21), *t*_₁/₂_ = 9.08 days (95% CI = 7.16–12.40 days) (*P* < 0.001).

**FIGURE 2:**
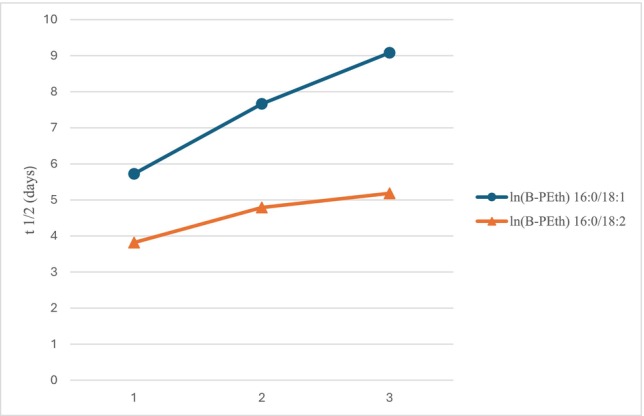
B‐PEth 16:0/18:1 and B‐PEth 16:0/18:2 mean half‐life (*t*_1/2_, days), by weeks 1–3 of abstinence. In abstinence week 4, B‐PEth was not present at levels high enough for interpreting half‐life at the subgroup level. B‐PEth = Phosphatidylethanol in blood; ln(B‐PEth) = logarithm of B‐PEth.

A similar pattern was observed for B‐PEth 16:0/18:2: week 1, *t*₁/₂ = 3.82 days (95% CI = 3.60–4.07 days); week 2, *t*₁/₂ = 4.79 days (95% CI = 3.97–6.03 days) (*P* = 0.003); week 3, *t*₁/₂ = 5.18 days (95% CI = 5.11–5.26 days) (*P* < 0.001) (Figure 2; Tables 3 and 5–6).

**TABLE 5:** Summary of individual half‐lives (*t*_1/2_, days) of B‐PEth 16:0/18:1 and B‐PEth 16:0/18:2 during 3 weeks of abstinence^a^.

	*n*	Mean (SD); min.–max.	Median	CV (%)
ln(B‐PEth) 16:0/18:1	78	6.75 (1.47); 3.67–10.36	6.62	21.82
ln(B‐PEth) 16:0/18:2	77	4.24 (0.69); 2.90–5.87	4.16	16.33

Abbreviations: B‐PEth = phosphatidylethanol in blood; CV = coefficient of variation; ln(B‐PEth) = logarithm of B‐PEth; *n* = number of subjects; SD = standard deviation.

^a^ Including subjects with sufficient data.

**TABLE 6:** First‐order kinetics of B‐PEth 16:0/18:1 and B‐PEth 16:0/18:2 half‐lives (*t*_1/2_, days), by week of abstinence^a^.

Variable	By week of abstinence	*n*	*t*_1/2_ (days) (95% CI)
ln(B‐PEth) 16:0/18:1	Week 1 of abstinence (days 1–7)	99	5.72 (5.30–6.22)
	Week 2 of abstinence (days 8–14)	56	7.67 (6.08–10.38)
	Week 3 of abstinence (days 15–21)	58	9.08 (7.16–12.40)
ln(B‐PEth) 16:0/18:2	Week 1 of abstinence (days 1–7)	99	3.82 (3.60–4.07)
	Week 2 of abstinence (days 8–14)	56	4.79 (3.98–6.03)
	Week 3 of abstinence (days 15–21)	58	5.18 (5.11–5.26)

Abbreviations: B‐PEth = phosphatidylethanol in blood; ln(B‐PEth) = logarithm of B‐PEth; *n* = number of subjects.

^a^ At abstinence week 4 (days 22–28), interpreting half‐life at the subgroup level was not possible.

Individual half‐lives during 3 weeks of abstinence were calculated using linear regression and differed slightly from the overall model estimates: for B‐PEth 16:0/18:1 (*n* = 78), the mean *t*_₁/₂_ = 6.75 days (range = 3.67–10.36 days; median = 6.62 days; SD = 1.47 days; coefficient of variation, CV = 21.82%); and for B‐PEth 16:0/18:2, the mean *t*₁/₂ = 4.24 days (range = 2.90–5.87 days; SD = 0.69 days; CV = 16.33%) (Table 7).

**TABLE 7:** Test of difference between B‐PEth half‐lives (*t*_1/2_, days), by week of abstinence.

	Week of abstinence	*P*
ln(B‐PEth) 16:0/18:1	Week 1 (days 1–7) vs week 2 (days 8–14)	0.002*
	Week 1 (days 1–7) vs week 3 (days 15–21)	<0.001*
	Week 2 (days 8–14) vs week 3 (days 15–21)	0.18
ln(B‐PEth) 16:0/18:2	Week 1 (days 1–7) vs week 2 (days 8–14)	0.003*
	Week 1 (days 1–7) vs week 3 (days 15–21)	<0.001*
	Week 2 (days 8–14) vs week 3 (days 15–21)	0.32

Abbreviations: B‐PEth = phosphatidylethanol in blood; ln(B‐PEth) = logarithm of B‐PEth. At week 4 of abstinence (days 22–28), interpreting half‐life at the subgroup level was not possible.

**FIGURE 3:**
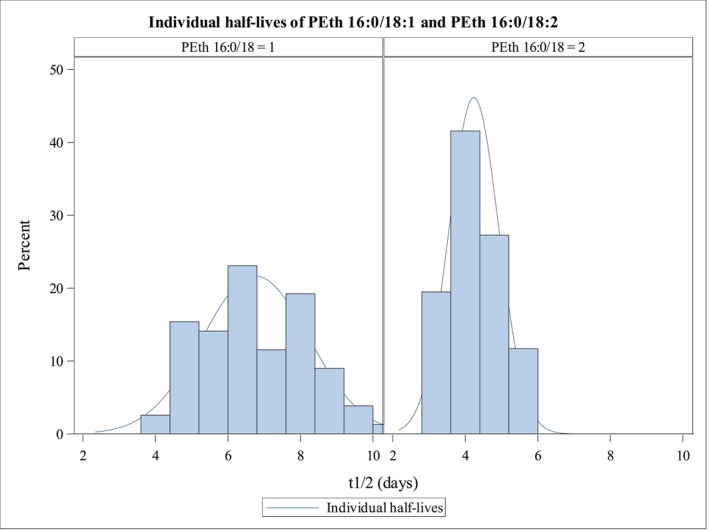
Histogram of individual half‐lives (*t*_1/2_, days) of B‐PEth 16:0/18:1 and B‐PEth 16:0/18:2 during the first week of abstinence. B‐PEth = phosphatidylethanol in blood.

These individual variations suggest interpersonal differences in B‐PEth decline, which were not explained by sex (Figure 3).

Spearman’s rank correlation analysis showed a low correlation between self‐reported alcohol consumption in the 4 weeks before abstinence and B‐PEth levels. B‐PEth 16:0/18:1 was significantly correlated with self‐reported intake in the first, second and third weeks before abstinence, with the highest correlation (*r* = 0.25, *P* = 0.014) in the second week before abstinence. The correlation for alcohol consumption 4 weeks before abstinence was considerably lower.

## DISCUSSION

Self‐reported alcohol consumption remains a common basis for both clinical and research decisions [24, 25]. However, the objective alcohol biomarker B‐PEth is gaining global recognition. While B‐PEth is well documented as a superior alcohol marker [1, 2], further research is needed to understand the factors influencing its decline—particularly in abstinent patients, as this is often a key consideration in clinical and legal settings.

This study, which examines a large sample of patients with AUD over a 3‐week period of abstinence, supports previous findings regarding B‐PEth half‐life. Additionally, it provides new insights into the influence of abstinence duration on B‐PEth levels, the optimal time frame for accurate B‐PEth detection and potential sex differences. The study also examines the correlation between objective alcohol markers and self‐reported alcohol consumption.

At the beginning of the study, patients had a mean B‐PEth 16:0/18:1 level (the most commonly analysed homologue) of approximately 2 μmol/l, a level consistent with severe AUD patients requiring withdrawal treatment. Self‐reported alcohol consumption in the week preceding treatment averaged at 211 g of alcohol per day (approx. 15 units of 14 g of 100% ethanol). The self‐reported alcohol intake increased over the weeks before withdrawal treatment. In the TLFB method, recall bias is reportedly larger the further from the consumption time point that the documentation is performed [26]. However, there is also commonly an escalation of alcohol intake in the weeks before admittance to alcohol withdrawal treatment, and we believe that this is the main cause of the escalation of reported alcohol intake.

B‐PEth elimination followed first‐order kinetics. The 16:0/18:1 homologue exhibited a longer half‐life than the 16:0/18:2 homologue, though the latter showed less individual variation. These results align with previous studies on B‐PEth half‐life for both homologues [15, 16, 17, 18]. The elimination rate slowed progressively each week after alcohol cessation, with no sex differences being observed. The degradation of B‐PEth in erythrocytes is not fully understood. Erythrocytes in blood have a lifespan of approximately 120 days, while the lifespan of B‐PEth in blood is significantly shorter, suggesting enzymatic degradation of B‐PEth. As B‐PEth is membrane‐bound, its availability for enzymatic degradation may decrease depending on the week of abstinence.

The B‐PEth levels remained detectable in the fourth week of abstinence, showing that the possibility of residual B‐PEth presence after 4 weeks of abstinence should be considered when assessing alcohol relapse after withdrawal treatment. The knowledge of individual variations in half‐life and detectable B‐PEth levels for many weeks is important to convey to clinicians when interpreting B‐PEth levels in relation to alcohol intake in patients. Repeated measurements and B‐PEth reduction may be used to verify abstinence even when B‐PEth values exceeds the limit of abstinence [27].

This study also reinforces that sex does not significantly impact B‐PEth formation at the level analysed. However, as B‐PEth formation depends on blood alcohol concentration (BAC), sex‐related differences in BAC [28] could affect B‐PEth production. Notably, individual variability was less pronounced for the 16:0/18:2 homologue, which is not currently the preferred marker in clinical practice. Co‐analysing both homologues might reduce variability and improve accuracy.

A consistently low correlation between B‐PEth levels and self‐reported alcohol consumption has been reported in previous studies, reflecting the limitations of subjective data. The results of this study show a significant but low (*r* = 0.25) correlation between self‐reported data and B‐PEth, with the highest correlation found in the second week before the start of abstinence. Recall accuracy is known to be stronger closer to the time of drinking [26], and both psychological or cognitive factors may explain the level of recall. The best time frame for collecting data can be of importance in interpreting subjective data, as self‐reported alcohol intake is still the prevailing method of data collection. However, the low correlation suggests inaccuracy in the self‐reported intake and advocates the use of objective alcohol markers in both clinical and research settings.

### Strengths and limitations

Although the sex ratio in this study reflects that of alcohol withdrawal care, it limits the full exploration of sex‐specific differences. The dropout rate was expected for this population, and the sample size was sufficient for statistical analyses. The lower *n* analysed for the fourth week of abstinence results from the shifting of sampling beyond the 21 days of abstinence in the protocol, and is not linked to dropout *per se*. However, the low number and the variations of individual values in the fourth week of the study period prevented further analysis of B‐PEth decline. Objective markers confirmed abstinence, and the inclusion of participants within 72 hours of alcohol cessation ensured a reliable baseline B‐PEth level. Although subjects were not hospitalised during the entirety of the study period, we believe that with the objective relapse markers for exclusion (U‐EtG, BrAC, B‐PEth elevation) and the ongoing treatment and follow‐up from the clinic, the probability of abstinence in the participants is very high. Eleven subjects showed positive drug screening despite substance use disorder being an exclusion criterion. Positive drug screening was, however, not an exclusion criterion and recreational drug use is not uncommon in this patient population, as is the use of prescription drugs. We do not consider the positive drug screening to have any effects on the study results. As PEth formation and elimination appear to be consistent across sexes and populations, the findings from this Swedish in‐patient sample are likely generalizable to most populations.

## CONCLUSION

This study on patients with AUD demonstrates that B‐PEth half‐life differs in the first week of withdrawal compared with the second and third weeks, with no significant sex differences. The overall mean half‐life, following first‐order kinetics, was 7.24 days for the 16:0/18:1 homologue and 4.55 days for the 16:0/18:2 homologue. The B‐PEth levels remains detectable in the fourth week of abstinence. Self‐reported alcohol intake shows a significant correlation with B‐PEth, with the highest, albeit low, correlation found 2 weeks before alcohol cessation.

## AUTHOR CONTRIBUTIONS

**Andrea de Bejczy:** Conceptualization (lead); data curation (equal); formal analysis (equal); funding acquisition (equal); investigation (equal); methodology (equal); project administration (equal); resources (equal); supervision (equal); validation (equal); visualization (equal); writing—original draft (lead); writing—review and editing (equal). **Lisa Walther:** Conceptualization (supporting); data curation (equal); formal analysis (equal); methodology (equal); resources (equal); validation (equal); visualization (equal); writing—original draft (supporting); writing—review and editing (equal). **Cecilia Nilsson‐Wallmark:** Conceptualization (supporting); data curation (equal); investigation (equal); methodology (equal); project administration (equal); supervision (equal); writing—original draft (supporting); writing—review and editing (equal). **Barbro Askerup:** Conceptualization (supporting); data curation (equal); investigation (equal); methodology (equal); project administration (equal); supervision (equal); writing—original draft (supporting); writing—review and editing (equal). **Anders Isaksson:** Conceptualization (supporting); data curation (equal); formal analysis (equal); methodology (equal); resources (equal); validation (equal); visualization (equal); writing—original draft (supporting); writing—review and editing (equal).

## DECLARATION OF INTERESTS

Andrea de Bejczy is co‐founder and co‐owner of Sobrera Pharma AB, Cecilia Nilsson‐Wallmark and Barbro Askerup are co‐owners of Sobrera Pharma AB, Lisa Walther and Anders Isaksson have no interests to declare.

## Supporting information


**Table S1** Description of excluded subjects during study period owing to violation of abstinence, categorised after the first excluding variable.
**Table S2** Self‐reported daily alcohol consumption by TLFB, 1–4 weeks before start of abstinence, in grams alcohol.

## Data Availability

The data that support the findings of this study are available from the corresponding author upon reasonable request.
