# Supplementary material for: Decline of phosphatidylethanol (B‐PEth) during abstinence in patients with alcohol use disorder undergoing withdrawal treatment, and the correlation of B‐PEth with self‐reported alcohol intake
Source: Addiction. 2026 Feb 19;121(6):1474–82. doi: 10.1111/add.70359 (PMC13155280; doi:10.1111/add.70359)
Supplement: Supplementary file 1 — Table S1 Description of excluded subjects during study period owing to violation of abstinence, categorised after the first excluding variable. Table S2 Self‐reported daily alcohol consumption by TLFB, 1–4 weeks before start of abstinence, in grams alcohol. [file ADD-121-1474-s001.docx]

**Table S1** Description of excluded subjects during study period due to violence of abstinence, categorized after the first excluding variable

| **Exclusion after** | **U-EtG** | ***Breath alcohol °Rising B-PEth** | **Self -reported alcohol intake (TLFB)** |
| --- | --- | --- | --- |
| Visit 2 | 3 |  | 2 |
| Visit 3 | 4 | 1* | 9 |
| Visit 4 | 2 | 1° | 2 |

U-EtG = Ethyl glucuronide in urine, B-PEth =Phosphatidylethanol in blood

**Table S2** Self-reported daily alcohol consumption by TLFB, 1- 4 weeks before start of abstinence, in grams alcohol

|  | **N** | **Grams of alcohol**  **Mean (SD); min- max** | **Median** |
| --- | --- | --- | --- |
| 1^st^ week prior to abstinence | 99 | 211 (110); 30.4-808 | 203 |
| 2^nd^ week prior to abstinence | 99 | 192 (109); 0-517 | 188 |
| 3^rd^ week prior to abstinence | 99 | 164 (117); 0-493 | 160 |
| 4^th^ week prior to abstinence | 96 | 152 (121); 0-493 | 126 |

N =number of subjects, SD = standard deviation, TLFB =Timeline Followback
